# Combination Therapy of Immune Checkpoint Inhibitors with Locoregional Therapy for Hepatocellular Carcinoma

**DOI:** 10.3390/cancers15205072

**Published:** 2023-10-20

**Authors:** Yasuyuki Tamai, Naoto Fujiwara, Takamitsu Tanaka, Shugo Mizuno, Hayato Nakagawa

**Affiliations:** 1 Department of Gastroenterology and Hepatology, Graduate School of Medicine, Mie University, Tsu 514-8507, Japan; tamai304051@clin.medic.mie-u.ac.jp (Y.T.); tanakatakamitsu@med.mie-u.ac.jp (T.T.); nakagawah@med.mie-u.ac.jp (H.N.); 2 Department of Hepatobiliary Pancreatic and Transplant Surgery, Graduate School of Medicine, Mie University, Tsu 514-8507, Japan; mizunos@clin.medic.mie-u.ac.jp

**Keywords:** hepatocellular carcinoma, immune checkpoint inhibitor, locoregional therapy, cancer–immunity cycle, clinical trial

## Abstract

**Simple Summary:**

Immune checkpoint inhibitor (ICI) therapy has recently become the standard treatment for advanced hepatocellular carcinoma (HCC); however, clinical outcomes remain unsatisfactory. Locoregional therapies, such as ablation, transarterial embolization, and radiotherapy, which are usually used for local treatment of HCC at an earlier stage, have been actively explored to enhance ICI efficacy. This review focuses on the rationale and clinical trials of combination therapy with ICIs and locoregional therapy for HCC.

**Abstract:**

Hepatocellular carcinoma (HCC) is estimated to be the fourth leading cause of cancer-related deaths globally, and its overall prognosis is dismal because most cases are diagnosed at a late stage and are unamenable to curative treatment. The emergence of immune checkpoint inhibitors (ICIs) has dramatically improved the therapeutic efficacy for advanced hepatocellular carcinoma; however, their response rates remain unsatisfactory, partly because >50% of HCC exhibit an ICI-nonresponsive tumor microenvironment characterized by a paucity of cytotoxic T cells (immune-cold), as well as difficulty in their infiltration into tumor sites (immune excluded). To overcome this limitation, combination therapies with locoregional therapies, including ablation, transarterial embolization, and radiotherapy, which are usually used for early stage HCCs, have been actively explored to enhance ICI efficacy by promoting the release of tumor-associated antigens and cytokines, and eventually accelerating the so-called cancer–immunity cycle. Various combination therapies have been investigated in early- to late-phase clinical trials, and some have shown promising results. This comprehensive article provides an overview of the immune landscape for HCC to understand ICI efficacy and its limitations and, subsequently, reviews the status of combinatorial therapies of ICIs with locoregional therapy for HCC.

## 1. Introduction

Hepatocellular carcinoma (HCC), representing >80% of primary liver cancers, is estimated to be the fourth leading cause of cancer-related deaths worldwide [1]. While the early detection of HCC enables curative treatments, such as liver transplantation, surgical resection, and ablation therapies, that lead to prolonged survival [2,3,4,5], the most common HCC stage at diagnosis is an advanced stage that is not amenable to curative treatments in North America, Europe, China, and South Korea, resulting in poor prognosis (5-year survival < 15%) [6]. In addition, as evidenced by the observation that the major cause of death in patients with early-stage HCC is HCC itself due to highly frequent metachronous and multicentric recurrences in chronically injured livers [7], the majority of HCC can eventually progress to an advanced stage even when the primary tumor is diagnosed at an early stage and is curatively treated. Therefore, more efficacious treatments for advanced HCC are required to improve the overall prognosis for HCC patients.

Advancements in immune checkpoint inhibitors (ICIs) have revolutionized systemic antitumor therapy for a variety of malignancies, including HCC. Anti-programmed death ligand-1 (PD-L1) atezolizumab plus anti-vascular endothelial growth factor (VEGF) bevacizumab and anti-programmed cell death protein 1 (PD-1) camrelizumab plus anti-VEGF receptor 2 (VEGFR2) rivoceranib demonstrated prolonged overall survival and progression-free survival compared with sorafenib that had been the only therapeutic option for unresectable HCC since 2008 [8,9,10]. A combination regimen of anti-PD-L1 durvalumab and anti-cytotoxic T-lymphocyte antigen 4 (CTLA4) tremelimumab also demonstrated significantly improved overall survival compared with sorafenib, with a 3-year survival rate of 30.7% [11]. Consequently, multiple expert societies consistently recommend ICIs as first-line systemic therapy for unresectable HCC, unless contraindications are identified [12]. Furthermore, the use of ICI in adjuvant or neoadjuvant settings has also been intensively investigated. Numerous clinical trials are ongoing to test antibodies targeting other immune checkpoints, such as lymphocyte activation gene 3 (LAG3), T cell immunoglobulin mucin-3 (TIM3), and T cell immunoreceptor with lg and ITIM domains (TIGIT) [13,14,15]. Thus, ICIs will continue to be the primary therapeutic option for HCC over the next decade.

Nevertheless, their current response rates are limited, with an objective response rate of 20–30% in clinical trials and real-world studies [8,11,16,17]. On the other hand, it should also be emphasized that the survival curves for ICI therapy frequently exhibit a plateau at the tail end that has never been observed in those with traditional cytotoxic chemotherapies or molecular target agents, suggesting that ICIs can enable almost complete remission in a subset of patients with HCC and a long cancer-free status [18]. Therefore, maximizing the potential antitumor ability of ICIs in combination with other modalities/therapies may enable further improvement in patients with advanced HCCs. Recently, locoregional therapies, such as ablation, transarterial embolization, and radiotherapy, which are usually used for HCC at an earlier stage, have been actively explored to bolster ICI efficacy. In this article, we first provide an overview of the immune landscape for HCC to understand ICI efficacy and its limitations, and subsequently review the status of combinatorial therapies of ICI with locoregional therapies for HCC and their future prospects.

## 2. Tumor Immune Microenvironment for HCC and ICI Efficacy

**Figure 1:**
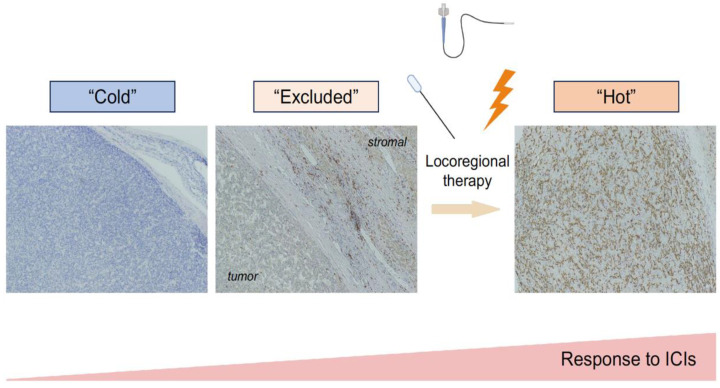
Expected response to immune checkpoint inhibitors (ICIs) in “hot”, “excluded”, and “cold” tumors. Brown (3,3′-diaminobenzidine (DAB)) staining represents CD8. These “hot”, “excluded”, and “cold” tumor phenotypes are determined according to the absolute abundance of T cells. The abundance of infiltrated immune cells that exert antitumor activity is expected to be positively correlated with susceptibility to ICI. Created with BioRender.com accessed on 18 October 2023.

Most carcinomas, including HCC, exhibit a heterogeneous pattern of immune cells within the tumor site, according to type, density, and localization. The widely accepted concept of immune heterogeneity associated with ICI efficacy classifies tumors into “hot”, “excluded”, and “cold” based on the T cell landscape (Figure 1). While immune-hot tumors have abundant T cells that exert antitumor activity and are, therefore, expected to be more susceptible to ICIs, immune-cold tumors are characterized by a paucity of T cells, likely resulting in a poor response. Immune-excluded tumors are expected to show an intermediate response to ICIs, because T cells are mainly observed at the edge of the tumor without being capable of infiltrating themselves. Indeed, this simplified but powerful concept predicted the treatment outcomes of ICIs for various malignancies [19,20,21]. Bagaev et al. developed conserved pan-cancer microenvironment subtypes from >10,000 transcriptome profiles and classified cancers into four types: immune-enriched/non-fibrotic (equivalent to hot), immune-enriched/fibrotic (equivalent to excluded), fibrotic, and immune-depleted (equivalent to cold) tumors [22]. Notably, only the immune-enriched/non-fibrotic subtype was associated with a favorable response to ICIs, suggesting that physical fibrotic barriers, that is, excluded status, can diminish ICI efficacy despite the accumulation of T cells. Based on this classification, one-fourth of HCCs were classified as the ICI-responsive immune-enriched/non-fibrotic subtype. Comprehensive immunohistochemistry-based assessment of HCC, including 919 regions from 158 HCC nodules, revealed that HCC can be classified into three subtypes based on the abundance of the immune cells (immune-high, -mid, and -low) and coexisting cell types. Immune-mid can be further subdivided into mid1 and 2, the former of which is characterized by enrichment of the granular formation and infiltration of mast cells and neutrophils, and the latter shows a more homogeneous distribution of various immune cell types. The immune-low subtype can also be subdivided into two distinct patterns, characterized by higher and lower regulatory T cell (Treg)/CD4 ratios. According to this histological classification, approximately 10% of HCC cases were classified as immune-high, which was significantly associated with a lower recurrence rate after surgical resection.

The underlying mechanisms and biomarkers of ICI effectiveness in HCC have been explored. Zhu et al. performed integrated molecular analyses of HCC samples (n = 358) from patients enrolled in the phase 1b or IMbrave150 phase 3 clinical trial of atezolizumab and bevacizumab to identify potential biomarkers for predicting clinical outcomes. Pre-existing immunity characterized by intratumoral CD8 T cell density, high expression of *CD274* encoding PD-L1, and T-effector signature was favorably associated with the outcome, whereas a high regulatory T cell to effector T cell ratio and high expression of oncofetal genes (*GPC3* and *AFP*) were associated with reduced benefit from the combination therapy. A variety of underlying mechanisms that promote T cell accumulation within tumors have been revealed in animal models and comprehensive human HCC analyses [23]. Ruiz de Galarreta et al. showed that β-catenin activation promotes immune escape by defecting dendritic cell recruitment and impairing T cell activity [24]. In steatotic HCC, palmitate-induced lipid accumulation upregulates PD-L1 expression and promotes immunosuppressive phenotypes of co-cultured macrophages and fibroblasts [25]. To explore the molecular correlates of response to ICIs among immune-hot HCCs, Magen et al. analyzed resected HCC samples from a neoadjuvant anti-PD-1 trial [26]. First, the authors confirmed that none of the immune-excluded or immune-cold HCCs responded to the ICI therapy. Simultaneously, the authors found that half of the immune-hot HCCs were nonresponsive. Multilayer omics analyses, such as single-cell RNA sequencing and spatial profiling, revealed that clonal expansion of a subset of T cells, more specifically intratumoral C-X-C chemokine ligand 13 (CXCL13)+ cholesterol 25-hydroxylase (CH25H)+ interleukin (IL)-21+ PD-1+ CD4 T helper cells named “CXCL13+ T_H_” and granzyme K (GZMK)+ PD-1+ CD8 effector-like T cells, was observed in responders, while terminally exhausted T cells were more enriched in non-responders. Interestingly, the potential antitumor CD8 effector-like T cells clonally expanded and differentiated from progenitor CD8 T cells in micro-niches formed by maturation regulatory dendritic cells and CXCL13+ T_H_ in response to ICI treatment. This was not observed in non-responders, indicating that it not only increases in CD8 T cells but also the creation of a favorable microenvironment before ICI initiation plays a pivotal role in ICI responsiveness.

### Rationale for Combination of ICI and Locoregional Therapies

**Figure 2:**
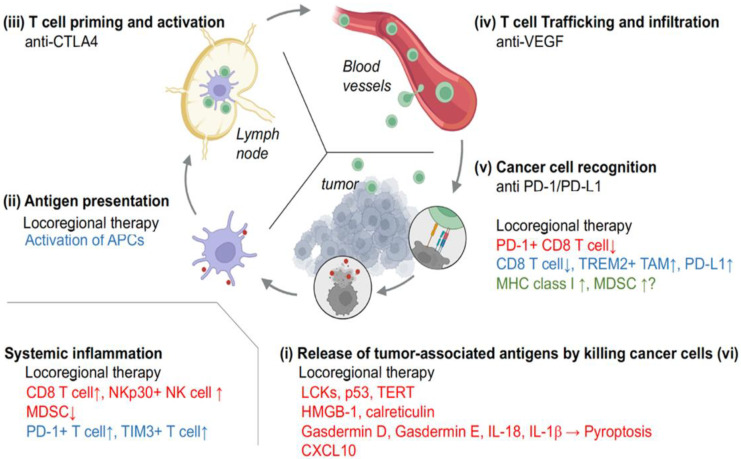
Immune responses against cancers after immune checkpoint inhibitors and locoregional therapy in the cancer–immunity cycle. The cancer–immunity cycle consists of six steps. The red, blue, and green molecules represent plausible molecular modifications by ablation, transarterial embolization, and radiotherapy, respectively. Created with BioRender.com accessed on 12 September 2023. LCKs, lymphocyte-specific protein tyrosine kinases; TERT, telomerase reverse transcriptase; HMGB-1, high mobility group box 1; IL, interleukin; CXCL10, C-X-C motif chemokine ligand 10; NK, natural killer; MDSC, myeloid-derived immunosuppressive cells; PD-1, programmed death-1; TIM3, T cell immunoglobulin mucin-3; APC, antigen-presenting cells; CTLA4, cytotoxic T-lymphocyte antigen 4; VEGF, vascular endothelial growth factor; PD-L1, programmed death ligand-1; TREM2, triggering receptor expressed on myeloid cells 2; TAM, tumor-associated macrophage; MHC, major histocompatibility complex.

As mentioned above, the efficient delivery and infiltration of specific T cells that exert antitumor activity at the tumor site are crucial for maximizing ICI efficacy. The rationales for multimodal approaches can be conceptualized by the so-called cancer–immunity cycle that illustrates the step-by-step anticancer immune responses and optimizes the combination therapies with ICIs accordingly (Figure 2) [27]. Briefly, the cancer–immunity cycle can be summarized as follows: (i) the release of cancer cell antigens, dying cancer cells release antigens (tumor-associated antigens (TAAs) and tumor-specific antigens aka neoantigen) that antigen-presenting cells (APCs), such as dendritic cells, recognize and engulf, as well as damage-associated molecular patterns (DAMPs) and cytokines; (ii) antigen presentation, APCs present tumor antigens on their surfaces; (iii) T cell priming and activation, T cells recognize tumor antigens presented by APCs through T cell receptors on their surface and are activated by recognition in the lymph node; (iv) T cell trafficking, activated T cells leave the lymph node towards the tumor site and infiltrate the tumor; (v) cancer cell recognition, T cells recognize and bind to cancer cells through interactions between the T cell receptors and cancer cell antigens present on the cancer cell surface; (vi) killing cancer cells, T cells release cytotoxic molecules, such as perforin and granzymes, to directly kill cancer cells, as well as cytokines and chemokines to activate other immune cells, such as macrophages. According to this concept, the anti-CTLA-4 antibody inhibits the immunosuppressive interaction between CTLA4 on T cells and APCs (step iii). Targeting VEGF with bevacizumab and rivoceranib is expected to increase cytotoxic immune cell infiltration into tumors by normalizing aberrant tumor vascularity, as well as other pathways (step iv). It is also important to evaluate how the combined therapy modifies the entire tumor microenvironment. We previously reported that anti-VEGF lenvatinib effectively recruits GZMK+ CD8 T cells to the tumor site through CXCL9 released from tumor-associated macrophages; however, it also enriched intratumoral stroma by upregulating fibrosis-related pathways [28].

In this scenario, dying cancer cells after locoregional therapies can release various immunogenic substances, such as TAAs and DAMPs, which may enhance local and systemic immune responses to ICIs. Zerbini et al. demonstrated that HCC-specific T cell responses were activated by coculturing patient monocytes in vitro together with tumor debris generated by radiofrequency ablation (RFA) and granulocyte macrophage colony-stimulating factor [29]. Importantly, apoptotic and necrotic cell death can induce different immune responses [30]. Antigen release by locoregional treatment can also expect the “abscopal effect” that leads to tumor shrinkage outside of tumors treated with locoregional treatment via the activated systemic immune response against tumors [31]. As overviewed below and illustrated in Figure 2, locoregional therapies mainly accelerate steps (i), (ii), (v), and (vi), as well as systemic inflammation. Given that the current ICI, with or without tyrosine kinase inhibitors (TKIs), primarily targets steps (iii) to (vi), combination therapies of locoregional therapies, ICI, and TKI are likely to activate the cancer–immunity cycle more efficiently. Indeed, the phase 3 EMERALD-3 trial is conducted to evaluate the efficacy and safety of durvalumab + tremelimumab + transarterial chemoembolization (TACE), with or without lenvatinib, compared with TACE alone (NCT05301842) [32].

## 3. Cancer–Immunity Cycle Acceleration by Locoregional Therapies

### 3.1. Ablation Therapy

Thermal ablation induces irreversible cell injury, tumor apoptosis, and coagulative necrosis through the local application of extremely high or low temperatures from electrodes that are directly inserted into tumors under image guidance. For early-stage HCCs, ablative techniques have been used for decades for the curative treatment of HCC. A multicenter randomized controlled study in Japan recently showed that percutaneous RFA is similarly effective for improving the prognosis of early-stage HCC compared to surgical resection [33]. Generally, ablation therapy can be classified into hyperthermal techniques, including RFA and microwave ablation (MWA), and cryoablation. RFA generates frictional heating (60–100 °C) through a high-frequency alternating current in the electrodes, whereas MWA generates heat using electromagnetic waves from an intratumorally placed antenna that forces the rotation of the molecules and increases their kinetic energy, thereby elevating the temperature within the tumor [34]. In contrast, cryoablation uses liquefied gas, such as argon, to reach a lethal cold temperature between −20 and −40 °C. These technical differences may lead to distinct immunogenicity, which can affect the effectiveness of combination therapy with ICIs [35]. Wang et al. performed in vitro experiments to compare the effects of different ablation temperatures (−80 °C, −40 °C, 0 °C, 37 °C, and 60 °C) on immunogenic cell death-related substances in multiple cell lines and found that the release of ATP, high mobility group box 1 (HMGB-1), and CXCL10 in HCC cell lines was significantly increased after both cryoablation and thermal ablation, whereas the expression levels of calreticulin, one of the DAMPs, were significantly different between high- and low-temperature ablation [36].

### 3.2. RFA

In a mouse HCC model, tumor ablation significantly increased and activated antigen-loaded dendritic cells that primed T cell activation in draining lymph nodes and, subsequently, activated tumor-specific T cells [37,38]. Mizukoshi et al. revealed that some peptides from TAAs, including lymphocyte-specific protein tyrosine kinases, p53, and human telomerase reverse transcriptase (TERT), are recognized by T cells only after locoregional treatment of human HCC [39]. Furthermore, the addition of the anti-CTLA4 antibody might enhance the immune response by modulating the cytokine and chemokine profiles of peripheral mononuclear cells. RFA may also induce pyroptosis, inflammasome-related programmed cell death, leading to the cleavage of gasdermin D and activation of IL-18 and IL-1β. Yang et al. showed that thermal sub-ablation of endothelial cells and hemangiomas induces HMGB-1-induced pyroptosis [40].

RFA also affects the systemic inflammatory status of patients with HCC. Myeloid-derived immunosuppressive cells (MDSCs) are a dominant component of the immunosuppressive network [41]. A pharmacological inhibitor in their differentiation from early-stage myeloid progenitors augmented the antitumor activity of ICIs in mouse models of mammary cancer [42]. The proportion of circulating MDSC was significantly decreased after RFA and was inversely associated with survival in patients with HCC [43]. RFA also triggers a systemic immune response, mainly involving innate immune cells, such as dendritic cells and natural killer (NK) cells, even after ablation of small HCC nodules in patients with cirrhosis [44]. An increase in activated NKp30+ NK cells 24 h after RFA was associated with reduced HCC recurrence, suggesting the importance of innate immunity in suppressing residual but clinically invisible malignant cells. RFA stimulated NK cell cytotoxicity and NK-mediated antibody-dependent cellular cytotoxicity. Furthermore, the production of nitric oxide (NO) and active oxygen species is activated after RFA [45]. NO induced the repolarization of tumor-associated macrophages to the tumoricidal form and the recruitment of NK cells and may complement ICI efficacy [46]. It is also known that sublethal heat treatment by RFA transforms HCC to the progenitor-like, more proliferative phenotype via epithelial–mesenchymal transition and promotes metastases associated with poor prognoses, supporting the importance of complete ablation for overall prognosis improvement [47,48].

### 3.3. MWA

In addition to heat-induced apoptosis and necrosis, Yu et al. speculated that MWA might induce ferroptosis, a newly proposed type of cell death, through the induction of reactive oxygen species, p53, the heat-shock protein, and NF-E2 related factor 2 (NRF2) [49]. Accumulating evidence suggests that ferroptotic cells attract and activate innate immune cells, such as neutrophils, and are efficiently engulfed by phagocytes [50]. Also, ferroptotic cells release HMGB-1, one of the well-known DAMPs, as a “find-me” signal, likely resulting in promoted immune cell recruitment [51]. If MWA truly induces ferroptosis, it might have another option to activate the immune response to ICIs.

### 3.4. Cryoablation

Cryoablation stimulated antitumor immunity together with immunoadjuvant therapy in a rat HCC model, leading to prolonged survival [52]. In a small series of 13 patients with unresectable liver tumors who underwent cryoablation, a subset of patients showed tumor necrosis, not only in directly ablated tumors but also in distant tumors, suggesting that cryoablation might induce the abscopal effect [53]. Furthermore, pretreatment serum tumor necrosis factor-alpha (TNF-α) and IL-10 levels were associated with the emergence of an abscopal effect. Zeng et al. showed that the proportions of PD-L1+ monocytes and PD-1+ CD8 T cells were positively correlated with the HCC stage [54]. Interestingly, the proportions were reduced after cryoablation, suggesting that cryoablation might modulate the systemic inflammatory status. Distinct immune modulation between cryoablation and hyperthermal techniques has yet to be confirmed in HCC. Inflammation induced by cryoablation may be greater than that induced by RFA in rat liver [55]. On the other hand, compared to RFA, cryoablation augmented both pro- and anti-inflammatory cytokines, including IL-1β, IL-5, IL-6, and IL-10, and did not reduce immunosuppressive regulatory T cells, although RFA reduced them, in a colon cancer mouse model [56].

### 3.5. Transarterial Embolization

Transarterial embolization therapy is the standard treatment for intermediate-stage HCC. HCC predominantly receives blood supply from the hepatic artery rather than from the portal vein, which is the major blood feeder for non-tumoral liver tissue. Therefore, the delivery of embolic agents, such as lipiodol, with or without anticancer drugs (TACE), drug-eluting bead (DEB)-TACE (DEB-TACE), or yttrium-90 (Y90)-loaded radioactive microspheres (transarterial radioembolization (TARE)), from a catheter placed in tumor-feeding arteries effectively leads to cancer lethality with minimal non-tumor liver injury. The hypoxic tumor state or cell death after transarterial embolization therapy is believed to drastically activate the intratumoral immune response through abundant antigen release. Differences in the mixed antitumor drugs or loaded materials may induce distinct immune responses. For TACE, doxorubicin or cisplatin is typically used to generate an emulsion with lipiodol. Cisplatin downregulates PD-L2 in human dendritic cells, whereas doxorubicin promotes immunogenic cell death and the clonal expansion of immunosuppressive MDSCs [57]. The type of embolic material also affects the magnitude of the immune response [58,59].

### 3.6. TACE/DEB-TACE

TACE appears to induce a systemic response in HCC by expanding AFP-specific CD4 T cells in the peripheral blood [60]. Tischfield et al. showed that the number of infiltrating CD3, CD4, and CD8 T cells, as well as the expression of PD-L1, was significantly increased in embolized tumors in a rat HCC model [58]. However, these findings remain controversial in human HCC. Consistent with the animal experiment, elevated PD-L1 expression was confirmed in human resected HCC after TACE [61]. However, another study demonstrated that CD4 and CD8 T cells, as well as immunosuppressive regulatory T cells, were reduced in human post-TACE HCCs compared to those without TACE [62]. To characterize the immune microenvironment post-TACE, Tan et al. subjected resected HCC samples with or without preoperative TACE to single-cell RNA sequencing [63]. Inconsistent with the above-mentioned rodent model observations, the authors found that the number of CD8 T cells was reduced after TACE compared to that in paired pre-TACE HCC tissues, whereas the triggering receptor expressed on myeloid cells 2 (TREM2)+ tumor-associated macrophages (TAMs), characterized by low expression of *CXCL9*, were increased. Mechanistically, TREM2^+^ TAM preferentially expressed galectin-1 encoded by *LGALS1*, which can hamper the functionality of CD8 T cells [64]. Moreover, galectin-1 induces the overexpression of PD-L1 in vascular endothelial cells, functioning as an obstacle to the migration of CD8 T cells into the tumor site. In two mouse HCC models, systemic TREM2 knockout enhanced the antitumor effect of the anti-PD-L1 antibody.

It is well known that malignant cells preferentially metabolize glucose through glycolysis to produce energy even under conditions of high oxygen, the so-called Warburg effect. Therefore, lactic acid concentration in malignancy is generally high, creating microenvironmental acidosis, although this may be etiology-dependent in HCC [65,66]. Tumor-derived lactic acid inhibits the migration of T cells and monocytes and facilitates immune evasion by tumors [67,68]. Conventional doxorubicin-mixed TACE normalizes the acidic environment in an animal model of HCC, which may lead to an ICI-susceptible microenvironment [69].

### 3.7. TARE

In the TARE procedure, Y90-loaded radioactive microspheres are transarterially injected into HCC and emit high-energy β-radiation to destroy tumor cells. As with conventional TACE and DEB-TACE, it has been reported that TARE can induce immunogenic cell death [70]. In addition, TARE has been shown to have a long-lasting antitumor effect (3–6 months) despite its short half-life (~60 h) [71,72]. Chew et al. showed that TARE activates the local and systemic immune response involving T cells, NK cells, natural killer T (NKT) cells, and APCs [73]. Interestingly, PD-1+ and TIM3+ CD8 T cells in peripheral blood from patients who showed sustained antitumor effect after Y90 TARE maintained their capability to express pro-inflammatory cytokines interferon-γ and TNF-α when stimulated ex vivo, suggesting that anti-PD-1 or anti-TIM3 ICIs after TARE, especially in sustained responders, may reactivate the antitumor effect as the sequential therapy. Indeed, an independent group showed that Y90 TARE followed by anti-PD-1 and anti-LAG3 inhibitors after one month induced synergistic immune-mediated HCC control [74]. Impaired lymphocyte function due to high radiation activity should also be considered as a side effect [75].

### 3.8. Radiotherapy

As the abscopal effect was originally reported as an unexpected regression in lesions outside the irradiated tumor in 1953 [76], radiotherapy can activate systemic and local immune responses by releasing antigens, DAMPs, and cytokines from dying cancer cells, as well as increasing major histocompatibility complex (MHC) class I on tumor cells [77]. Durvalumab has already been approved as a maintenance therapy after chemoradiation for stage III non-small cell lung cancer [78,79]. For HCC, Sung et al. developed a mathematical model to simulate the percentage of tumor volume irradiated to synergize antitumor ability with ICIs, and concluded that irradiating 90% of tumor cells in addition to ICIs yields an incremental benefit between 33% and 71% compared to that without irradiation [80]. Radiotherapy also triggers immune responses via gasdermin-E-mediated pyroptosis [81]. Furthermore, the cyclic guanosine monophosphate–adenosine monophosphate synthase (cGAS)-stimulator of interferon gene (STING) pathway has been reported to be the key to understanding radiotherapy-mediated immune responses. Du et al. reported that radiotherapy upregulated PD-L1 expression in HCC by activating the intrinsic cGAS-STING pathway, leading to immune evasion; therefore, combination therapy with anti-PD-L1 blockade potentiated the antitumor effect of radiotherapy [82]. Meanwhile, the activated cGAS-STING pathway in M1 macrophages promotes T cell recruitment to HCC, which may enhance ICI efficacy [83]. Anti-VEGF sorafenib may enhance the immune response to radiotherapy by downregulating the signal transducer and activator of transcription (STAT) pathways [84]. To induce these synergistic effects, gut dysbiosis may have to be modulated. Li et al. reported that cyclic di-AMP, an emerging second messenger of bacteria, serves as an agonist of STINGs and activates the cGAS-STRING-interferon I pathway, resulting in the suppression of antigen presentation and impairment of effector T cell functions [85]. During radiotherapy, organs outside the target tumor can be irradiated, which may lead to an immunosuppressive tumor microenvironment. Wang et al. reported that ionizing radiation induces immunosuppressive MDSC expansion, contributing to diminished antitumor immunity by ICIs through the YTH N6-methyladenosine RNA binding protein F2 (YTHDF2)-nuclear factor-kappa B (NFκB) axis [86].

### 3.9. In-Progress Clinical Trials of ICI Plus Locoregional Therapies

With these promising experimental and preclinical studies, dozens of early- to late-phase clinical trials investigating the combination therapies of ICIs and locoregional therapies are ongoing (Table 1).

#### 3.9.1. Ablation Therapy

A phase 2 clinical trial evaluated the combination of RFA and anti-CTLA-4 tremelimumab in 32 patients with Barcelona Clinic Liver Cancer (BCLC) B/C HCC (NCT01853618) [98]. No severe side effects were observed, and 26% of the patients showed a partial response (PR). Interestingly, tumor biopsies after six weeks showed increased CD8 T cells in patients showing a clinical benefit. Median time to tumor progression (TTP) and overall survival (OS) were 7.4 and 12.3 months, respectively. Another proof-of-concept trial (n = 50) also suggested that the addition of ablation therapy to anti-PD-1 pembrolizumab or nivolumab increased the objective response rate (ORR) (24% vs. 10% in combination therapy and anti-PD-1 monotherapy, respectively) and achieved a prolonged median OS (16.9 vs. 5.0 months, respectively) with acceptable toxicity profiles in patients with advanced HCC (NCT03939975) [90]. In the phase 2 IR11330 trial, 48 patients with advanced HCC after at least one line failed systemic therapy received subtotal ablation, defined as the complete treatment of 1–5 lesions by hyperthermal ablation and intentionally leaving residual lesions intact, followed by anti-PD-1 toripalimab initiation on days 3 or 14 after ablation therapy or toripalimab monotherapy (NCT03864211). The ORRs of ablation therapy plus toripalimab on days 3 or 14, and toripalimab monotherapy were 38%, 31%, and 19%, respectively [92].

Given the high frequency of recurrence, even after curative treatment, of early stage HCCs (76% and 70% 5 years after curative RFA and surgical resection, respectively) [111,112], adjuvant ICI therapies for at-risk patients after curative treatment have been intensively tested in prospective clinical trials, with an expected immune-activated state after curative treatment. The phase 3 IMbrave050 trial showed that adjuvant atezolizumab plus bevacizumab significantly improved recurrence-free survival (RFS) compared to active recurrence surveillance in patients with early but high-risk HCC after curative RFA and surgical resection (hazard ratio (HR), 0.72) (NCT04102098) [87]. Phase 3 CheckMate 9DX (NCT03383458), KEYNOTE-937 (NCT03867084) [88], and EMERALD-2 (NCT03847428) [89] trials are currently in progress to evaluate the efficacy of adjuvant nivolumab, pembrolizumab, and durvalumab in improving RFS after curative treatment in patients with early-stage HCC, respectively.

#### 3.9.2. TACE

To enhance the immune response against intermediate- and advanced-stage HCC, combination therapies of TACE and ICI have been investigated in prospective clinical trials. In a phase 1/2 clinical trial, TACE followed by anti-CTLA-4 tremelimumab had a higher PR rate and prolonged OS with acceptable adverse events (NCT01853618) [98]. Anti-PD-1 pembrolizumab following TACE achieved 10.8 months of progression-free survival (PFS) from the first TACE (NCT03397654) in the phase 1/2 PETAL trial [96]. The phase 2 IMMUTACE trial evaluating the effects of TACE in combination with nivolumab for intermediate-stage HCC resulted in a complete response (CR) and a PR of 16% and 55%, respectively. At a median follow-up of 20 months, the median PFS was 7.2 months (NCT03572582) [94,113].

#### 3.9.3. TARE

In a prospective open-label, phase 1 clinical trial of nivolumab plus Y90 TARE in patients with advanced HCC, 9 (81%) and 6 (46%) out of 11 patients exhibited stable disease and reduced serum AFP levels, respectively (NCT02837029) [103]. In a phase 2 trial in which 40 patients underwent Y90 TARE plus nivolumab, one (3%) and ten (28%) patients showed CR and PR, respectively (NCT03033446) [101]. A phase 2 NASIR-HCC trial evaluated the combination of TARE and nivolumab in 42 patients with unresected HCC and demonstrated an objective response rate of 42% and a median TTP and OS of 8.8 and 20.9 months, respectively (NCT03380130) [100]. A prospective multicenter single-arm phase 2 clinical trial investigated pembrolizumab plus Y90 TARE in patients with HCC that was likely refractory to TARE alone, defined as a multifocal disease, branch portal vein thrombosis, and/or diffuse distribution (NCT03099564) [102]. Despite the refractory nature, the median PFS, TTP, and OS were 8.6, 9.9, 22.0 months, respectively.

#### 3.9.4. Stereotactic Body Radiotherapy (SBRT)

In a multicenter phase 1 clinical trial, patients with advanced or unresected HCC received either nivolumab alone or nivolumab plus ipilimumab, followed by SBRT. Clinical outcomes favored the nivolumab plus ipilimumab arm compared with nivolumab alone, with an ORR of 57% (four of seven patients) and 0% (zero of six patients), a median PFS of 11.6 and 2.7 months, and a median OS of 41.6 versus 4.7 months, respectively. In the combined immunotherapy group, the 3-year survival rate was 57%, with an acceptable safety profile (NCT032033040) [108]. The single-arm phase 2 START-FIT trial investigated sequential TACE and SBRT followed by anti-PD-L1 avelumab in patients with locally advanced hepatocellular carcinoma who were unsuitable for curative treatment (NCT03817736) [109]. Among the 33 enrolled patients, 14 (42%) and 4 (12%) achieved radiological CR and curative treatment, respectively. In a single-arm trial including 21 patients with unresectable HCC, SBRT plus camrelizumab demonstrated a median PFS and OS of 5.8 and 14.2 months after a median follow-up of 19.7 months, respectively [114].

## 4. Conclusions

The emergence of ICIs has dramatically and rapidly changed the therapeutic landscape for malignancies, including HCC. As summarized, ICIs will be used for early- to late-stage HCC, given their broad antitumor mode of action. Therefore, an in-depth understanding of ICIs from bench to bedside is required to effectively realize their potential. To further maximize the synergistic ability of ICIs and locoregional therapy, the treatment sequences and timing must be optimized. Immune-enhancing strategies, such as cancer vaccination and nanomedicine via HCC-targeting peptides, may also improve ICI efficacy [115,116]. In addition, biomarkers for identifying patients who would benefit the most from combination therapies are an unmet need to efficiently use limited medical resources [117]. The accumulating evidence indicates that immune evasion plays a pivotal role, not only in fully developed HCC, but also in de novo HCC development from premalignant lesions, suggesting that combination therapy may be immune preventive [118,119]. Elucidating these unanswered questions will eventually improve the prognosis for HCC patients. We are witnessing the dawn of a revolution in cancer therapy using ICIs.

## Figures and Tables

**Table 1:** In-progress clinical trials of combination therapies of locoregional therapies and ICIs.

Clinical Trial Identifier (Study Acronym) *	Study Phase	^#^Pt.	Locations	Eligible HCC Stage	Combined ICI and TKI	ORR	DCR	Median PFS, RFS	OS	**TRAE ^†^**	**Clinical Trial Status** **(As of 15 September 2023)**	**Reference**
**Ablation** **(or surgical resection)**												
NCT04102098 (IMbrave050)	3	668	Global	Adjuvant	Atezolizumab + bevacizumab	-	-	22.1 m	-	41.0%	Active, not recruiting	[87]
NCT03383458 (CheckMate 9DX)	3	545	Global	Adjuvant	Nivolumab	-	-	-	-	-	Active, not recruiting	
NCT03867084 (KEYNOTE-937)	3	950	Global	Adjuvant	Pembrolizumab	-	-	-	-	-	Active, not recruiting	[88]
NCT03847428 (EMERALD-2)	3	908	Global	Adjuvant	Durvalumab +/− bevacizumab	-	-	-	-	-	Active, not recruiting	[89]
NCT04727307 (AB-LATE02)	2	202	France	Neoadjuvant	Atezolizumab + bevacizumab	-	-	-	-	-	Recruiting	n.a.
NCT03939975	2	50	China	BCLC B/C	Pembrolizumab or nivolumab or toripalimab	24.0%	68.0%	5.0 m	16.9 m	14.0%	Completed	[90]
NCT03753659 (IMMULAB)	2	30	Germany	BCLC A	Pembrolizumab	-	-	17.4 m	-	-	Active, not recruiting	[91]
NCT03864211 (IR11330)	1/2	145	China	BCLC C	Toripalimab	31.2–37.5%	-	-	-	25.0%	Active, not recruiting	[92]
NCT04652440	1/2	30	China	BCLC A/B	Tislelizumab	-	-	-	-	-	Recruiting	n.a.
**TACE**												
NCT04340193 (CheckMate 74W)	3	26	Global	BCLC B/C	Nivolumab + ipilimumab	-	-	-	-	-	Active, not recruiting	n.a.
NCT04712643 (TALENTACE)	3	342	China, Japan	BCLC B/C	Atezolizumab + bevacizumab	-	-	-	-	-	Active, not recruiting	[93]
NCT05301842 (EMERALD-3)	3	725	Global	BCLC B/C	Durvalumab + tremelimumab +/− lenvatinib	-	-	-	-	-	Recruiting	[32]
NCT04268888 (TACE-3)	2/3	522	United Kingdom	BCLC B	Nivolumab	-	-	-	-	-	Recruiting	n.a.
NCT03572582 (IMMUTACE)	2	49	Germany	BCLC B	Nivolumab	71.4%	75.5%	7.2 m	28.3 m	34.7%	Completed	[94]
NCT04814030 (AIPD1)	2	80	China	BCLC C	Sintilimab	-	-	-	-	-	Recruiting	n.a.
NCT05751343	2	55	China	BCLC B/C	Atezolizumab + bevacizumab	-	-	-	-	-	Recruiting	n.a.
NCT03638141	2	30	United States	BCLC B	Durvalumab + tremelimumab	-	-	-	-	-	Recruiting	n.a.
NCT04997850	1/2	142	China	BCLC B/C	Camrelizumab or sintilimab + lenvatinib	78.9%	94.4%	17.1 m	-	53.5%	Enrolling by invitation	[95]
NCT03397654 (PETAL)	1/2	26	United Kingdom	BCLC B	Pembrolizumab	-	-	10.8 m	-	21.0%	Active, not recruiting	[96]
NCT03143270	1	20	United States	BCLC B	Nivolumab	22.2%	100.0%	-	-	-	Active, not recruiting	[97]
**Ablation and TACE**												
NCT01853618	1/2	61	United States	BCLC B/C	Tremelimumab	26.3%	-	7.4 m	12.3 m	-	Completed	[98]
NCT04220944	1	45	China	BCLC B/C	Sintilimab	-	-	-	-	-	Recruiting	n.a.
**TARE**												
NCT05377034 (STRATUM)	2	176	Singapore	BCLC B/C	Atezolizumab + bevacizumab	-	-	-	-	-	Recruiting	n.a.
NCT05063565 (ROWAN)	2	100	United States, Spain	BCLC B/C	Durvalumab + tremelimumab	-	-	-	-	-	Recruiting	[99]
NCT03380130 (NASIR-HCC)	2	41	Spain	BCLC B/C	Nivolumab	41.5%	92.7%	9.0 m	20.9 m	21.4%	Completed	[100]
NCT03033446	2	40	Singapore	BCLC B/C	Nivolumab	30.6%	-	-	-	6.0%	Active, not recruiting	[101]
NCT03099564	1	30	United States	BCLC B/C	Pembrolizumab	27.0%	84.7%	8.6 m	22.0 m	-	Active, not recruiting	[102]
NCT04605731	1	32	United States	BCLC B/C	Durvalumab + tremelimumab	-	-	-	-	-	Recruiting	n.a.
NCT02837029	1	27	United States	BCLC B/C	Nivolumab	-	82.0%	-	-	-	Completed	[103]
NCT03812562	1	2	United States	Neoadjuvant	Nivolumab	-	-	-	-	-	Active, not recruiting	n.a.
**SBRT**												
NCT04167293 (ISBRT01)	2/3	116	China	BCLC C	Sintilimab	-	-	-	-	-	Recruiting	n.a.
NCT04913480	2	37	Hong Kong	BCLC B/C	Durvalumab	-	-	-	-	-	Recruiting	n.a.
NCT03857815	2	30	China	BCLC B	Sintilimab	96.0%	-	-	-	4.0%	Recruiting	[104]
NCT05396937	2	42	China	BCLC C	Atezolizumab + bevacizumab	-	-	-	-	-	Recruiting	[105]
NCT03316872	2	30	Canada	BCLC B/C	Pembrolizumab	-	-	-	-	-	Recruiting	n.a.
NCT05286320	1/2	27	Taiwan	BCLC B/C	Pembrolizumab + lenvatinib						Not yet recruiting	n.a.
NCT04857684	1	20	United States	Neoadjuvant	Atezolizumab + bevacizumab	-	-	-	-	-	Recruiting	n.a.
NCT05185531 (Notable-HCC)	1	20	China	Neoadjuvant	Tislelizumab	-	-	-	-	-	Recruiting	[106]
NCT05096715	1	20	United States	BCLC B/C	Atezolizumab + bevacizumab	-	-	-	-	-	Not yet recruiting	n.a.
NCT05488522	1	18	United States	BCLC B/C	Atezolizumab + bevacizumab	-	-	-	-	-	Recruiting	[107]
NCT03203304	1	14	United States	BCLC B/C	Nivolumab + ipilimumab	57.1%	-	11.6 m	41.6 m	61.6%	Terminated	[108]
**TACE and SBRT**												
NCT03817736 (START-FIT)	2	33	Hong Kong	BCLC B/C	Avelumab	42.4%	-	-	-	33.3%	Active, not recruiting	[109]
NCT04988945	2	33	Hong Kong	BCLC B/C	Durvalumab + tremelimumab	81.3%	-	-	-	31.3%	Recruiting	[110]

* Data were obtained from ClinicalTrials.gov (https://clinicaltrials.gov/) accessed on 15 September 2023. ^#^Pt., the number of patients. ^†^ Percentages of grade 3 or 4 treatment-related adverse events are shown. ICI, immune checkpoint inhibitor; HCC, hepatocellular carcinoma; TKI, tyrosine kinase inhibitor; ORR, objective response rate; DCR, disease control rate; PFS, progression-free survival; RFS, recurrence-free survival; OS, overall survival; TRAE, treatment-related adverse event; BCLC, Barcelona Clinic Liver Cancer; n.a., not available.
